# Antifragile and Resilient Geographical Information System Service Delivery in Fog Computing

**DOI:** 10.3390/s22228778

**Published:** 2022-11-14

**Authors:** Tahira Sarwar Mir, Hannan Bin Liaqat, Tayybah Kiren, Muhammad Usman Sana, Roberto Marcelo Alvarez, Yini Miró, Alina Eugenia Pascual Barrera, Imran Ashraf

**Affiliations:** 1 Department of Computer Science, University of Gujrat, Gujrat 50700, Pakistan; 2 Department of Information Sciences, University of Education, Lahore 41000, Pakistan; 3 Department of Computer Science (RCET), University of Engineering and Technology, Lahore 54890, Pakistan; 4 Department of Information Technology, University of Gujrat, Gujrat 50700, Pakistan; 5 Higher Polytechnic School, Universidad Europea del Atlántico, Isabel Torres 21, 39011 Santander, Spain; 6 Project Management, Universidad Internacional Iberoamericana, Arecibo, PR 00613, USA; 7 Department of Project Management, Universidade Internacional do Cuanza, Cuito, Bié, Angola; 8 Department of Project Management, Universidad Internacional Iberoamericana, Campeche 24560, Mexico; 9 Fundación Universitaria Internacional de Colombia, Bogotá 111311, Colombia; 10 Department of Information and Communication Engineering, Yeungnam University, Gyeongsan 38541, Korea

**Keywords:** fault tolerance, fog computing, cloud computing, geographical information systems, fragility resilience

## Abstract

The demand for cloud computing has drastically increased recently, but this paradigm has several issues due to its inherent complications, such as non-reliability, latency, lesser mobility support, and location-aware services. Fog computing can resolve these issues to some extent, yet it is still in its infancy. Despite several existing works, these works lack fault-tolerant fog computing, which necessitates further research. Fault tolerance enables the performing and provisioning of services despite failures and maintains anti-fragility and resiliency. Fog computing is highly diverse in terms of failures as compared to cloud computing and requires wide research and investigation. From this perspective, this study primarily focuses on the provision of uninterrupted services through fog computing. A framework has been designed to provide uninterrupted services while maintaining resiliency. The geographical information system (GIS) services have been deployed as a test bed which requires high computation, requires intensive resources in terms of CPU and memory, and requires low latency. Keeping different types of failures at different levels and their impacts on service failure and greater response time in mind, the framework was made anti-fragile and resilient at different levels. Experimental results indicate that during service interruption, the user state remains unaffected.

## 1. Introduction

Cloud computing (CC) has earned excellent admiration and popularity over the past few years for processing and storing big data. It follows a pay-as-you-go model which renders services and resources on request over the Internet. CC is fabricated in advanced data centers with interrelated servers and can host a vast number of applications. The data centers are based on virtualized computing resources which are delivered to the user in the form of virtual machines (VMs). Mobility, scalability, and reduced usage costs are its prominent benefits. However, CC is prone to failure due to factors such as unreliable software/hardware, natural adversities, and human-made faults [1]. To overcome the shortcomings associated with CC, fog computing (FC) has received huge attention. FC has a distributed infrastructure in which cloud services are delivered and extended near the edge of the network [2].

FC is a virtualized platform that has been proposed to provide computing at edge devices that can deliver new applications and services for future applications [3]. Future Internet is dependent on improved quality of service (QoS) and quality of experience (QoE), which can be achieved through orchestrated services by reducing latency with high mobility, improved scalability, and real-time execution. The centralized architecture of the cloud and the stochastic nature of the Internet are barriers in way of delivering real-time services in the Internet of things (IoT). As a result, FC is brought into consideration as an intermediate layer between IoT and cloud for better provision of services [4].

In the FC paradigm, multiple fog nodes participate as clusters to perform processing. The user is one or two hops away from the fog node through a wireless connection, and the cloud is at a multi-hop distance. This distance produces higher latency in CC as compared to FC, and the cloud is unable to provide real-time interaction [5]. FC provides client data offloading efficiently in the minimum time by the seamless fusion of cloud and edge resources. It delivers better networking, storage, and management between edge and cloud. The CC architecture is centralized infrastructure, and FC is a decentralized infrastructure through a wireless connection. Therefore, the rate of failure is higher in FC as compared to CC. In contrast to the FC, CC offers QoS and fault tolerance, but due to its context-unawareness, it suffers latency while deploying services with high computational requirements [6]. Technical differences between CC and FC are shown in Table 1.

The term "anti-fragile" is fundamentally different from resilience, which is explained as "the ability to recover from loss or harm within the minimum possible time," and anti-fragility is to prevent the harm from happening in advance. Anti-fragile and resilient systems learn from failures to handle issues. Systems respond by a better approach in response to failures and improve operations concerning time. Resilience is to keep the system working and responding even in the presence of the fault and try to recover in the minimum possible time. Its main purpose is to maximize system availability by minimizing downtime.

In geographical information systems (GIS), a vast amount of geospatial data is retrieved, stored, and analyzed from multiple sources for end-users [7]. FC has become an emerging solution that can handle increased throughput while providing the low-power node and reducing latency at the client layer near the edges of numerous geospatial systems. FC utilizes less transmission power and less storage as compared to the cloud for indelible analysis of data [8]. Various emerging applications are based on CC-based frameworks, such as healthcare, watershed management, land use, coastal, marine, and urban planning. This framework has the capability of analyzing and integrating heterogeneous thematic layers for analyzing and creating alternate scenarios for geospatial data for several functions, such as over-relay analysis, statistical computing, data visualization, and query formation [8]. In a traditional CC-based GIS framework, geospatial data are processed and analyzed by the cloud, which requires high Internet bandwidth and extensive processing time. FC resolves this problem with the provision of local computation proximate to the client near the edge [9].

Smart applications—smart healthcare, smart homes, smart grids, environmental monitoring, etc.—are widely involved in today’s daily life. These applications require low latency, real-time processing, location-based services, and local storage. CC-based frameworks produce delay, bandwidth overhead, network congestion, and poor QoS. In FC, edge devices are distributed, latency-sensitive, and location-aware, and they have real-time service requirements. GIS is becoming popular and gaining a vibrant role in the provision of these applications in daily life. GIS requires reduced latency, real-time processing, and increased throughput while processing huge amounts of geospatial data and requires local storage.

The fault tolerance (FT) aspect of CC has been investigated widely, but FT in terms of resiliency and anti-fragility in service delivery in FC is still in its infancy; both need to be studied and investigated in FC [5]. There are many research issues and challenges associated with FC that require extensive research, such as scheduling, resource allocation, fog-based microservices, security, resiliency, privacy, and FT. There is a need for the incorporation of resiliency and anti-fragility in FC for ensuring uninterrupted services. Both currently need to be studied and investigated in FC [5]. GIS has been deployed as an application platform to utilize its services regardless of its FT aspect. This gave us the motivation for designing an FT framework in FC for the provision of GIS services to the user in an anti-fragile and resilient manner.

The anti-fragility and resilience in the designed framework have been incorporated for uninterrupted service delivery. This framework has been designed for a 3-layer architecture of FC. Peering fog nodes at the fog layer have been deployed to maintain resiliency. VMs are running in both of these nodes, and VMs are running Q-GIS service containers. The containers are provisioning services in a resilient manner. We have deployed this framework in a real environment as a test bed. The anti-fragility and resiliency are maintained at four different levels: container level, VM level, node level, and site level. Results prove that in case of failure at any of these levels, it would not interrupt the user.

The rest of the paper is organized as follows. The literature related to the current study is presented in Section 2. The proposed framework and its working are explained in Section 3. Section 4 presents the results and discussions, and the study is concluded in Section 5.

## 2. Literature Review

Several novel services have emerged and become an essential part of human life over the past decade, including smartphone technology, location-based services, smart health services, etc. To provide such services, a larger ICT setup is required that acts as a backbone. Geographically located huge ICT infrastructures are now in place with better and stable technology solutions to serve uninterrupted services. Huge ICT infrastructures have different dependencies in terms of physical resources and technological resources with respect to sustainability and stability. Different approaches are used in this regard, including proactive approach, self-healing, failure prediction, preemptive migration, etc.

The proactive approach, also called failure avoidance, monitors system behavior by applying multiple techniques. Failure prediction is mostly achieved through statistical modeling. The main techniques in this approach are prediction, monitoring, and reallocation of system resources [10]. Self-healing is the ability of a system to recover from faults by applying specific recovery procedures. A self-healing FT system depends upon various fault aspects, such as duration, location, and intensity [11].

**Figure 1:**
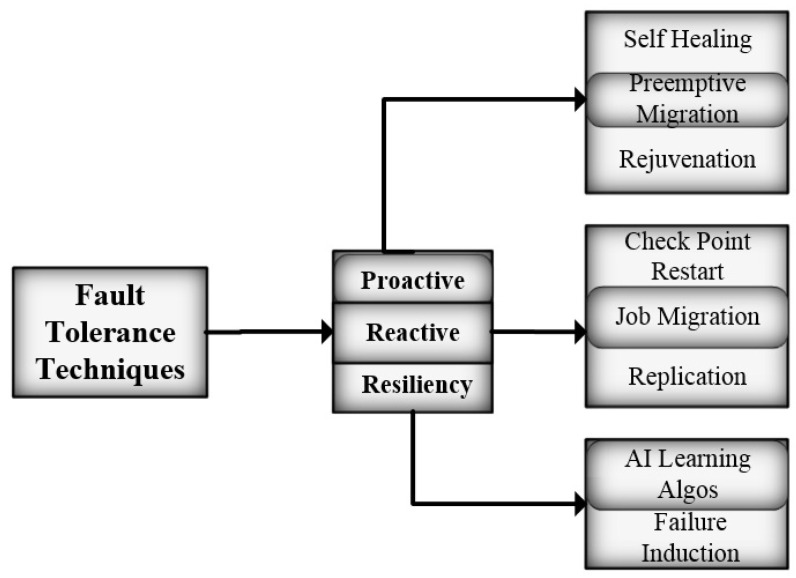
Fault tolerant approaches.

In preemptive migration, the task is migrated or offloaded from a suspicious node to some other node. Pre-fault indicators are used to predict the chance of occurrence of a fault on a specific node and timeframe. In many reviews and surveys regarding FT in CC software rejuvenation have been mentioned as proactive FT techniques [11,12,13,14]. In the software rejuvenation process, periodic backups of a system are taken. Rejuvenation is categorized as full rejuvenation and partial rejuvenation, depending on the components of the cloud environment [11]. The authors of [15,16,17] have discussed software rejuvenation techniques to avoid outage of cloud services. Figure 1 shows the classification of fault-tolerant techniques.

SHelp was introduced as a proactive FT framework using self-healing [18]. The authors later improved the work by introducing rescue points named ASSURE [19]. PFHC was introduced as a proactive FT framework by combining multiple proactive techniques for high-performance computing (HPC) [20]. WSRC was introduced for a cloud VM machine manager (VMM) using a software rejuvenation technique in variable time [21]. SRFSC was introduced using the software rejuvenation technique [22]. FTDG was proposed for stream computing as an FT scheduling framework [23].

Reactive FT techniques are applied after the occurrence of the fault. They do not pose any system overhead, as the system behavior is not monitored continuously. Multiple reactive approaches have been discussed in CC in the FT management scenario. In checkpoint/restart policies, the system state is saved on periodic intervals which may be from 60 s to 1024 s. If a fault occurs in a system, the system is restored from the previously known state [24]. The system starts from the last known state rather than from the beginning [11]. These stats are suitable for long-running jobs [10]. When some resource/node is failed, the job is migrated to another node. It is unlike preemptive migration, as the migration takes place after the occurrence of the failure. This approach is applied after a certain number of attempts of restart policy [11]. It is commonly used as a reactive technique in the CC FT paradigm [13]. HAProxy is one such example that uses job migration [12].

In replication, the task is replicated on several instances of VMs synchronously or asynchronously [25]. In this FT mechanism, at least one replica is placed in some other cluster to minimize the application failure [24]. Two types of failures have been widely discussed in the literature, active replication and passive replication [11]. BlobCR was introduced as a two-way checkpoint restart mechanism for infrastructure as a service (IaaS) for clouds using snapshots [26]. Later, this work was improved by using live incremental snapshotting [27].

BFTCloud framework was proposed for FT management in CC based on the replication technique [28] and was extended with minor modifications by [29]. The AASIF framework was proposed in CC based on the FIFO approach by serving nodes [30]. CAMAS was proposed for Amazon Cloud for FT management based on five checkpoints and migration techniques [31]. FTM was proposed for FT management for the IaaS cloud in which the FT service is supposed to be delivered by a third party for users as fault tolerance as a service (FTaaS) [24]. An FT framework was proposed for reliable cloud application services based on reactive FT mechanism [32]. FLBAFTM was proposed for FT management in the cloud for minimizing the probability of system failure by implementing reactive FT techniques [33].

Resilient methods combinations of proactive and reactive fault-tolerant methods with the ability to learn from the environment. Reactive methods take corrective measures after a fault occurs [18,19,24], and proactive methods try to maintain the resiliency of the service and offer better responsiveness [34]. Previous research has mostly focused on proactive and reactive FT mechanisms [32,33], but lately, resilient methods have been introduced as emerging FT solutions [10]. These methods have mainly been categorized into two categories. Machine learning provides a smart way to ensure FT and can be extended to CC and FG paradigms. The triangle approach, distributed dynamic queue, unified reinforcement learning (URL), ordinal sharing learning (OSL), and Markov decision process are some examples [10]. The failure induction technique is to manually insert the failure into the system to observe the system outage.

Researchers [29,35,36] have focused on fault tolerance in architecture-specific solutions in enterprise cloud infrastructures. The authors offered modular solutions in [37], which are based on combinations of distinguishable activities such as replication, detection, and recovery. The Spine-leaf FC Network has been proposed for the scalability of IoT data centers by controlling congestion [38].

Some studies have focused on scheduling for work offloading in neighboring nodes of FC [39]. Vehicular ad hoc networking based on software-defined networking in FC has been discussed [40]. Smart transportation based on vehicular ad hoc networks in FC has been proposed [41]. IoT-enabled healthcare systems have been provisioned by CC services for data analysis, scalability, and reliability. The data collected by sensors are transmitted for processing through multi-hop distance. This results in delay and adversely affects the processes/services which are latency sensitive; therefore, a fog based healthcare solution has been proposed [42]. Delay, privacy, and all these points urge towards offloading application segments to edge nodes/fog nodes which are located nearer to the edge/user devices [43]. Table 2 summarizes the related work in FT in CC.

## 3. Materials and Methods

The emergence of IoT has caused massive data explosions due to the interconnection of multiple ubiquitous devices, and these data cause network congestion. Cloud computing is becoming insufficient to fulfill the requirements for real-time, mobility-aware, geo-distributed, and latency-sensitive applications. In some situations, it is not suitable to send all data to the cloud for processing and storage to avoid bandwidth overhead. To overcome this situation, the FC has been introduced to process the data near the user. FT ensures the system’s availability in case of any failure. These failures may be hardware, software, network, or system failures.

In this work, a framework has been designed and deployed as a testbed with the incorporation of antifragility and resilience to guarantee service delivery. The quantum GIS (QGIS) is deployed as a service to be delivered through this framework. The primary objective of this framework is to monitor the status of the service and nodes on which it is deployed, and it should always be available to users. Any type of failure will not affect the service delivery to the user. The resilience in the proposed framework is maintained at four different levels: container level, VM level, fog node level, and site level.

### 3.1. Framework Methodology

**Figure 2:**
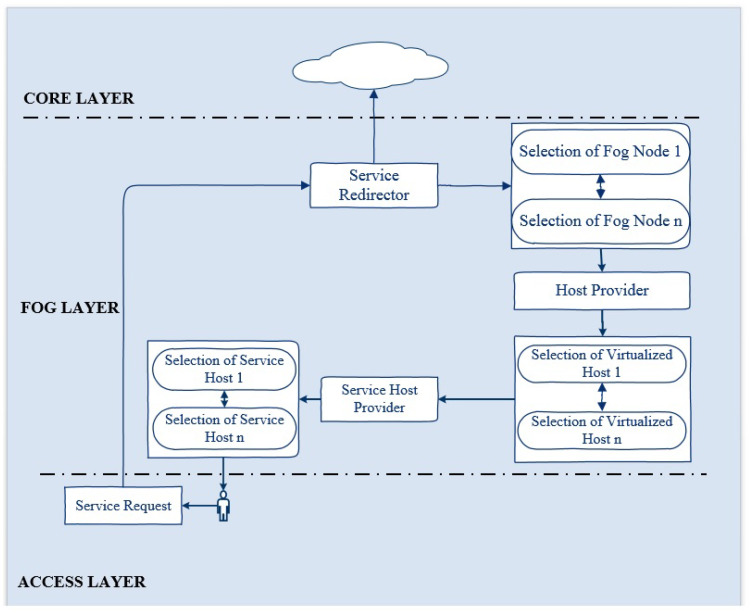
Workflow of the proposed framework.

Figure 2 illustrates the complete mechanism of the proposed framework. At the edge layer, the user requests a GIS service. This service is redirected to the first available fog node. The fog node routes the request to the main manager node (VM) which is responsible for maintaining the anti-fragility and resilience of the system. It redirects the request to the service host provider, which is running with a QGIS service in a Docker container. The processing of the fog node ensures the immediate response to a service request from the edge in the minimum time. Each of the fog nodes is running four VMs under a hypervisor. The manager in the fog node redirects the service request to the first available node (VM). If this node fails, the manager immediately routes the service request to the second available node (VM) in the minimum time. If this node fails or becomes overloaded, the manager redirects the service available to the third available node.

In the same way, if this node goes down or becomes overloaded, the manager immediately routes the service request to the fourth available node. Due to some reason, if this node becomes unavailable or gets over-occupied, the service will be automatically redirected to the second available fog node in a minimum time without affecting the user’s state while maintaining antifragility and resilience. This redirection would be seamless for the user. The fog node also has a manager node, which is responsible for forwarding the service request to the first available node, which is the VM running with QGIS service in the Docker container. As for fog node 1, if this serving node becomes inaccessible, the service will be automatically routed to the second available node. If this node becomes overloaded, the service will be redirected automatically to the third available node. If this node becomes unreachable due to any reason, the request will be routed to the last available node in fog node 2. If the last node (VM) becomes inaccessible or goes down or becomes over-occupied, the service will be redirected in minimum time to the core layer without interrupting the user state. In the core, we are running two instances with a QGIS service. The first available instance will entertain the service request. If this instance becomes unavailable or gets out of reach, the second instance will finally fulfill the user’s request without affecting the user.

### 3.2. Components of ARSDFC Framework

The proposed framework is based on 3 layers and includes major components, including the service request (SR), service redirector ($SR_{d}$), host selector (HS), virtual machine monitor (VMM), virtualized host (VH), and service selector (SS). Figure 2 illustrates the interactions among these components. Before going into their details, we briefly discuss these components. SR is initiated at the edge layer by the user to $SR_{d}$. $SR_{d}$ first calculates the state $S_{i}$ (i=1 to *m*, where *m* is the total number of sites) of all sites and then redirects it towards the first available site at the fog layer. The $SR_{d}$ forwards $SR_{n}$ to the core layer in case of the unavailability of sites at the fog layer. HS is the selector residing in $S_{i}$ and estimates the state $l_{j}$ (j=1 to *n*, where *n* is total number of VHs) of VHs and forwards SR to the best available VH. SS running inside the VH estimates the state $p_{k}$ (k=1 to *q*, where *q* is the total number of service containers (SC)) and assigns the available SC to SR.

#### 3.2.1. Service Request

The user initiates SR for QGIS hypertext transfer protocol (HTTP) service using transmission control protocol/Internet protocol (TCP/IP) or user datagram protocol (UDP) connection through his web browser. The data is transferred in the form of plain text between the user and the web browser. When a user requests an HTTP service through a TCP/IP connection, the session is established at the transport layer between the client and the web server when it replies to the user’s request. UDP is ideal for latency-sensitive applications such as online gaming and video streaming. The SR is established through TCP/IP via Ethernet switch or UDP via any wireless access point. The number of service requests is denoted by $SR_{n}$.

#### 3.2.2. Service Redirector

$SR_{d}$ receives $SR_{n}$ and calculates the most suitable $S_{i}$ using a round Robin domain name system (RR-DNS) in time $t_{r}$, where $t_{r}$ (r=1 to *w*, where *w* is the time of last SR) is the arrival time of SR. If a server providing service goes down, it redirects the SR to other available servers after calculation $SR_{d}$ assigns $SR_{n}$ to $S_{i}$. $SR_{d}$ assigns SR per site as follows.

(1) $$S_{i}=\frac{SR_{n}}{S-1}$$

In case of failure of sites at the fog layer, SR would be directed towards the core layer. $SR_{d}$ is defined as

(2) $$SR_{d}=\frac{SR_{n}}{S}$$

#### 3.2.3. Virtual Machine Monitor

Every $S_{i}$ is running with a hypervisor which is called a virtual machine monitor (VMM). A VMM is an emulator which shares the host’s resources such as CPU and memory across multiple VMs as the guest operating system. There are two types of hypervisor: bare metal, which runs directly on the hardware, and hosted hypervisor, which runs as a software layer on the OS. Hyper-V is a virtualization product by Microsoft which is provided as an optional feature in Windows server 2016. It is responsible for creating and managing VMs across the system. The bare-metal Hyper-V is deployed at each site. The Hyper-V is not FT itself, and it only hosts the guest VMs. The failover cluster for the Hyper-V has been configured to maintain the anti-fragility at the fog layer. Active directory services are configured, which authenticates the user and prevents unauthorized access to network resources. This means if the host site is down due to any reason, it would not affect the serving VMs at this site. Control of VMs in the failed sites will be shifted to another site, without affecting the VM’s state. The site residing in the failover uses a heartbeat of 5 s, which is defined under IEEE standard 1278. Sites in the fail-over cluster use the periodic signal of 5 s to synchronize with each other to show their normal operation. However, if a site does not respond within 5 s, the cluster assumes that the site has failed and shifts the control to other sites without interrupting the serving VMs in the failed site. This heartbeat rate has been denoted as $t_{s}$, where $t_{s}=5$ s.

#### 3.2.4. Host Selector

The host selector is the managing node, which monitors the state of the VMs deployed in the cluster and schedules the SR to the most suitable VH running inside the cluster. Traditional scheduling algorithms do not consider the timeline and mobility of the application while managing its resource allocation. User location should be monitored before applying resource allocation in FC for the avoidance of facing minimum delay in service delivery [50]. The scheduling decision in FC directly influences the data transmission over the network. A modified weighted round Robin has been implemented. We considered processor and memory status for placement of SR to the appropriate VH. There is one managing node $M_{n}$ at each site $S_{i}$, where i=1 to *m*, which is defined as

(3) $$M_{n}=\Sigma_{l=1}^{n}VH_{j}*\frac{S}{S-1}*2$$

where $\Sigma_{l=1}^{n}VH_{j}$ is the total number of virtual machines at each site $S_{i}$ and *S* is the total number of sites. The $M_{n}$ is responsible for the assignment of service to VH. It receives the SR and estimates the state of the VH by checking the threshold $\mu_{ij}$ for memory and CPU. The value of threshold $\mu_{ij}$ is between 0 and 1, and we have fixed it to 0.5.

(4) $$\mu_{ij}=\frac{\mu_{i}+\mu_{j}}{2}$$

The threshold for memory is represented by $\mu_{i}$ and for CPU $\mu_{j}$. To check the state of resources of VH before the assignment of the SR, the value of this threshold $T_{i}$ is calculated by the equation below.

(5) $$T_{i}=-mu_{ij}$$

where $T_{i}$ is the threshold of a VH for SR assignment, and the sum of states of all VHs is defined as

(6) $$VL=\sum_{l=1}^{n}l_{j}$$

where *j* defines the number of VHs at one site, and the load per VH is calculated as

(7) $$LPVH=\frac{VL}{l_{j}}*T_{i}$$

VL is the sum of all states of VH, $l_{j}$ is the state of an individual VH, and $M_{n}$ assigns the SR to VH only if

(8) $$LPVH<T_{i}$$

The $M_{n}$ present at $S_{i}$ checks the condition for SR assignment for all VHs running inside it. The VH that fulfills this condition starts delivering services to the user. If all VHs in one site fail to fulfill this condition, SR is redirected towards $SR_{d}$. This $SR_{d}$ will calculate the next $S_{i}$ through Equation (1).

The $M_{n}$ running in the next $S_{i}$ will select the VH fulfilling the condition defined in Equation (8). If all VHs running inside this site fail to fulfill this condition, defined in Equation (8), SR would be directed towards $SR_{d}$.

#### 3.2.5. Service Selector

SS is running inside each VH, where it monitors and manages the state $p_{k}$ (k=1 to *q* where *q* is the total number of containers) of the SCs running inside it. It calculates the state $p_{k}$ and assigns SR to it. The status of the SC is represented by θ, where the value of θ is (0 vs. 1). The number of states of SC is denoted by *q*. SR assignment to VH is defined as.

(9) $$SSC=\sum_{k=1}^{q}p_{k}$$

SSC is the sum of states of $p_{k}$, and *n* is the total number of SCs. SR to an SC would be assigned as follows

(10) $$SPC=\frac{SSC}{n}*\theta$$

where SPC is the service per container. If SPC=1, then service would be assigned; otherwise, SPC would be redirected towards HS.

### 3.3. Algorithm

In this section, Algorithm 1 is discussed in detail. The service request SR is initiated by the user using TCP/IP or UDP through any web browser. SR is redirected to the first available site $S_{i}$ at the fog layer at line number 3 using Equation (1). $SR_{d}$ receives $SR_{n}$ and calculates the most suitable $S_{i}$ using a round Robin domain name system (RR-DNS) in time $t_{r}$, where $t_{r}$ (r=1 to *w*, where *w* is the time of last SR 263) is the arrival time of SR. If a server providing service goes down, it redirects the SR to other available servers after calculation $SR_{d}$ assigns $SR_{n}$ to $S_{i}$. If the fog layer is unavailable or down, the service is redirected towards the core layer at line 5 using Equation (2).

The host selector is the managing node, which monitors the state of the VMs deployed in the cluster and schedules the SR to the most suitable VH running inside the cluster. Traditional scheduling algorithms do not consider the timeline and mobility of the application while managing its resource allocation. User location should be monitored before applying resource allocation in FC for the avoidance of facing minimum delay in service delivery. The scheduling decision in FC directly influences the data transmission over the network. A modified, weighted round Robin has been implemented. We considered processor and memory status for placement of SR to the appropriate VH. There is one managing node $M_{n}$ at each site $S_{i}$, where i=1 to *m*. Each site contains the managing node $M_{n}$. This managing node was calculated using Equation (3), which selects the first available virtual host VH by calculating the load per virtual host LPVH for memory and CPU using Equation (7) and comparing it to the threshold $T_{i}$, whose value should be between 0 and 1 using Equation (9). SR is assigned to VH only if the condition is fulfilled in Equation (9). Service selector SS is running inside each VH, which monitors the state of service container SC using Equation (10), which is 0 or 1. If this is true, the service is assigned to the user; otherwise, it is redirected toward the next VH.
**Algorithm 1:** Anti-fragile and resilient service delivery in fog computing.**Initialization**: SR⟵−11:**for**$S_{i}$ from 1 to *n* **do**2:   **if** true **then**3:     Calculate using Equation (1);4:   **else**5:     Calculate using Equation (2);6:     **for** $VH_{j}$ from 1 to *n* **do**7:        **if** true **then**8:          Calculate $M_{n}$ using Equation (3);9:          Calculate VL using Equation (6);10:        Calculate LPVH using Equation (7);11:        **if** Equation (9) true **then**12:             **for** SSC *k* from 1 to *q* **do**13:               Calculate using Equation (10);14:               **if** Equation (10) true **then**15:                  Return true;16:               **end if**17:             **end for**18:          **end if**19:        **end if**20:     **end for**21:   **end if**22:**end for**

### 3.4. Experimental Setup

The design of ASRDFC comprises three layers: core, fog, and edge layer. Each layer further comprises two layers: the hardware layer and the software layer.

#### 3.4.1. Core Layer

The core layer is based on cloud infrastructure which is deployed at the data center (DC). The infrastructure includes computing, storage, and network devices that are required to run cloud applications and services.

The hardware layer provides the physical infrastructure. Physical infrastructure includes the hardware required for running the cloud. Two Dell power edge R710 server machines are deployed as cloud servers. The Dell power edge R710 machine is a powerful server with good features incorporated, including high memory capacity, high CPU speed, substantial storage, and enhanced network I/Os. It contains multiple generations of Intel Xeon Quad-core processors with 2.26 GHz speed along with DDR3 RAM, which facilitates higher bandwidth and better power consumption. The 32 GB RAM for fog servers was deployed to enhance service delivery and availability. Each server has 1 terabyte (TB) of storage. The servers are operating with a redundant array of independent disk (RAID) configurations. The Huawei Quidway 7706 core switches were deployed for the network. These switches provide GBit/s access to wireless and wired network devices throughout the network. The details of hardware deployed in the framework are provided in Table 3.

The software layer is responsible for the virtualization of the hardware and provision of the applications and services by the cloud. A list of software components used in this framework is provided in Table 4.

- Linux/Ubuntu was deployed as an OS for managing the hardware and the software. Ubuntu was chosen because it is an open-source OS, and it provides a high level of security as compared to other operating systems.
- The spike version of OpenStack has been deployed as a cloud operating system to manage and control computing, storage, and network resources for the cloud.
- The kernel-based virtual machine (KVM) has been used as the hypervisor for the cloud. KVM is an open-source hypervisor and allows the running of multiple isolated instances of the OS, which are called virtual machines. KVM has efficient support for hardware, security, scheduling, scalability, and live migration, which makes it preferable as compared to other hypervisors.
- This OpenStack is deployed along with Nova, JSON, and Neutron APIs for computing, storage, and network resource management.
- Two virtual instances were created as part of the proposed framework.

**Figure 3:**
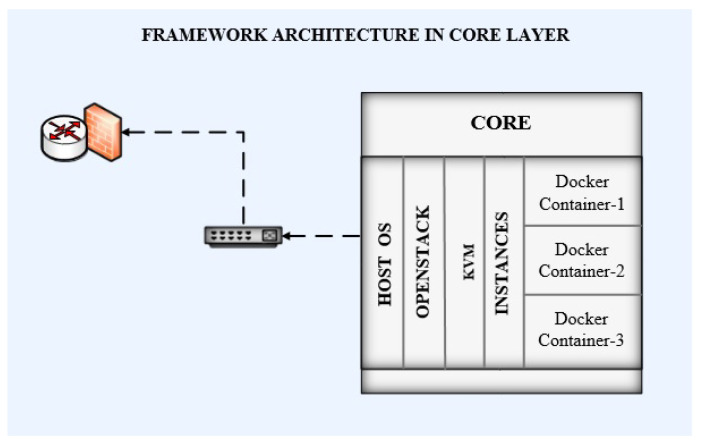
Architecture of core layer.

Framework architecture in the core layer contains a RAID. The RAID has been configured on both servers of the Dell power edge R710 using a RAID controller. Ubuntu OS has been installed as the serving host OS on these servers. The spike version has been used for the deployment of OpenStack. Nova, Neutron, and JSON APIs have been configured for managing computing, storage, and network resources. KVM has been used as the hypervisor for virtualization. Two instances of computing have been configured for VMs. Ubuntu has been deployed on both of these VMs. Docker Engine 16.1 has been configured for the management of Docker containers on these VMs. The image of the QGIS application was created to convert it into a Docker container. These containers have been deployed on the VMs. The VMs have been assigned IP addresses from the 10.1.2.0/24 subnet. Containers use the bridged IPs of their native VMs. The complete architecture at the core layer is shown in Figure 3. Openstack is running at the core layer with KVM. Each instance inside KVM is running service containers. The core is connected through an Ethernet switch to the router along the firewall. The core layer is working upon the fog layer as a backup.

#### 3.4.2. Fog Layer

The fog layer is responsible for the aggregation of the cloud services near the user. It is an extension of the cloud, which brings the processing of the core nearer to the user. The servers operating at this layer are called fog nodes/fog servers.

For the hardware layer, two Dell power edge R710 server machines are deployed as fog nodes, which are the same as cloud machines. The server contains an Intel Xeon quad-core processor with 32 GB of DDR3 RAM, and each server has 1 TB of storage using RAID configuration. Additionally, two networks’ I/Os are used to communicate with different networks. Furthermore, Huawei S5700 network switches are deployed for connectivity to the core and LAN. These switches provide GBit/s access to wireless and wired network devices connected throughout the network. For malware and intrusion detection, NSA 4600 Dell Sonic Wall is used to prohibit unauthorized access to our network. The detail of hardware deployed at the fog layer is shown in Table 3.

Fog nodes are virtualized devices running with multiple instances of VMs under a hypervisor. The tenant application processes are virtualized. They are isolated by fog software infrastructure and communicate with the help of an API. The details of the software deployed at the fog layer are provided in Table 4.

- Windows server 2016 is deployed on fog nodes due to enhanced security, affordable storage, improved intrusion detection, and virtualization protection.
- Clusters of fog nodes are configured with the help of Hyper-V.
- Ten instances of VMs in the Hyper-V cluster were created, and the Ubuntu OS has been deployed on each of these VMs. Linux/Ubuntu is utilized as it is an open-source OS with enhanced security and portability.
- Containers provide the abstraction of the applications which are running in an environment. Containers provide the virtualization of the OS. OS processes and their dependencies are isolated in OS virtualization, and these are managed by the OS kernel. Some examples of containers are LXC, BSD jails, Docker, Solaris Zones, and LXD. Docker 16.1’s container is deployed for our service. The software that hosts these containers is called Docker Engine, and the 12.1 version is used for the proposed framework.
- QGIS is a free and open-source program for using GIS functions. It can be installed on multiple platforms. GIS applications allow for dealing with spatial information. We converted it into a Docker image to be deployed as a container across the platform.
- Microsoft virtual machine converter (MVMC) is a tool supported by Microsoft for the conversion of VMware virtual machines into Hyper-V virtual machines. Initially, the framework in the VMware environment is used but later migrated to the Hyper-V platform.
- WinSCP is a free FTP and SFTP client for Windows to copy files between the host and remote system using a GUI.
- Putty terminal SSH client for windows is used to support SSH, SCP, Telnet, rsocket, and rlogin connections. Additionally, this program is used to connect with VMs deployed across the framework.
- Zabbix is an an-open source monitoring solution for networks, applications, and services. It offers reports and visualization of data stored across the network. Its reports, statistics, and configuration parameters are available through a web-based interface. The Zabbix server is deployed for framework monitoring and the Zabbix agent for QGIS service analysis on the client side.

**Figure 4:**
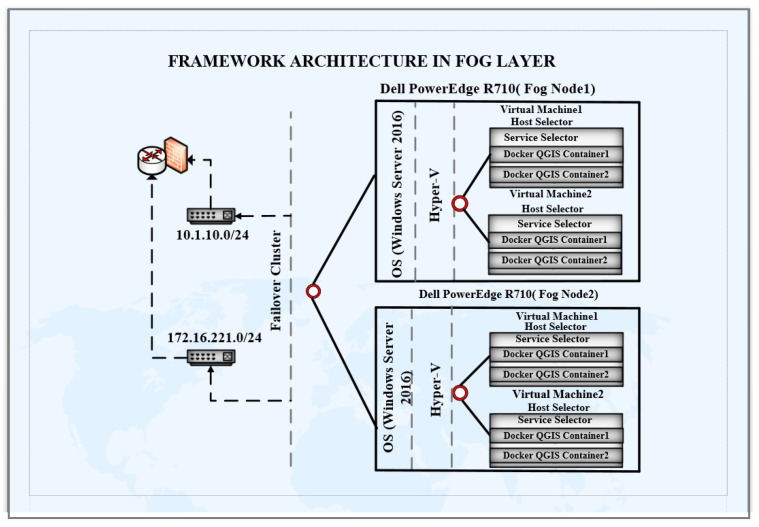
Architecture of fog layer.

As for the cloud infrastructure, the framework architecture in the fog layer uses two Dell power edge R710 machines as fog servers. RAID has been configured on both of these servers with 1 TB of storage on both server machines. Windows Server 2016 has been deployed as the host OS on both of these servers. Hyper-V has been configured on each of these machines. Hyper-V Failover Cluster has been configured for these servers. One server has been assigned an IP address from the 10.1.10.0/24 subnet. Additionally, another server has been assigned an IP address from the 172.16.221.0/24 subnet. These subnets have been configured in different virtual local area networks (VLAN). Eight VMs have been configured with four VMs on each of these server machines. Docker Engine 16.1 has been configured on each of these VMs, and the QGIS service is converted into a Docker container. Additionally, this Docker container has been deployed on each of the VMs instances. These containers use a bridge IP network with their VMs. The complete architecture has been shown in Figure 4.

#### 3.4.3. Edge Layer

The edge can be the users or any devices—cell phones, laptops, tabs, etc., which utilize the services from the fog layer. The details of hardware and software are mentioned in Table 3 and Table 4, respectively.

In the hardware layer, the service can be accessed using any laptop, iPhone, or Android phone with wireless or wired Internet connectivity. For the software layer, the service can be accessed over Wireless-LAN and LAN using any browser—i.e., Internet Explorer, Edge, Mozilla Firefox, Safari, and Google Chrome access the service over HTTP.

**Figure 5:**
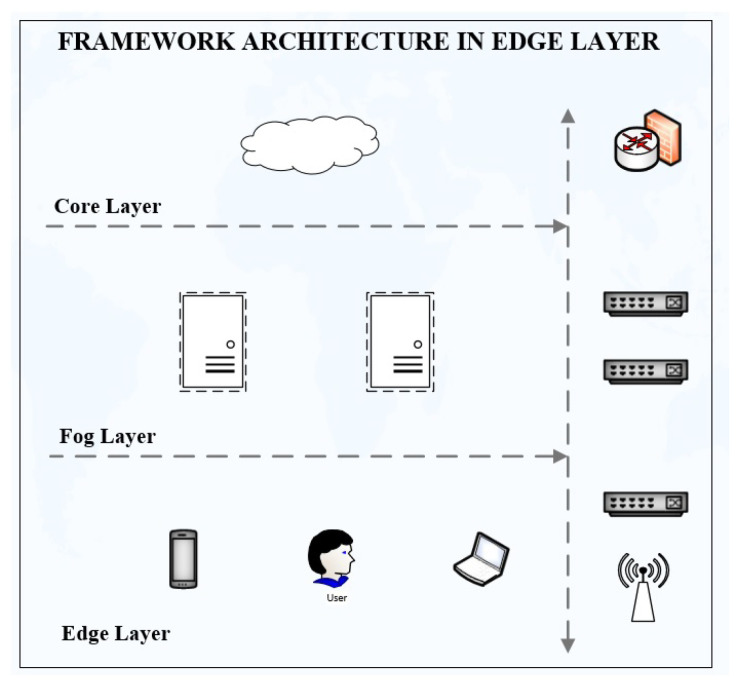
Architecture of edge layer.

The service has been tested over wireless LAN using TP-Link and Microtik (MT) wireless routers and wired connection via network switches on different browsers, such as Mozilla Firefox, Google Chrome, Edge, Internet Explorer, and Safari. Figure 5 shows the complete architecture of service requests at the edge layer. The user initiates service requests at the edge layer, which is routed to the fog layer by an Ethernet switch/wireless router. The fog layer is an intermediate layer between the edge and core layer.

#### 3.4.4. Framework Architecture

**Figure 6:**
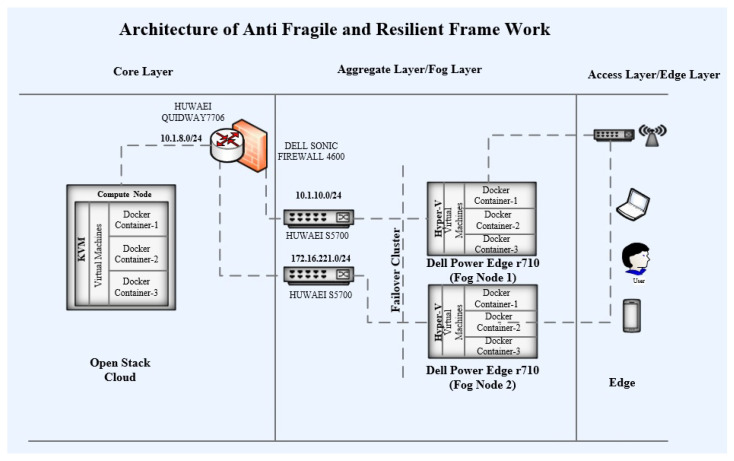
Architecture of the anti-fragile and resilient framework.

The designed system is used to deliver the services to the user in an anti-fragile and resilient manner, so that if there is any type of failure in the system at any level, it should keep performing and delivering the services to the user without any interruption. The failures may be hardware, software, and network failures; and an anti-fragile resilient framework was designed to tackle these issues. The framework has been designed for FC consisting of three layers, including the core, fog, and edge layers. The proposed framework maintains anti-fragility and resiliency at four different levels: the SC level, VH level, node level, and site level. Figure 6 shows the detailed physical and internal architecture of the proposed framework.

#### 3.4.5. Network Setup

For connecting the 3-layered architecture of the framework, the network is divided into different subnets residing in different VLANs. Cloud servers have been assigned IP addresses from the 10.1.8.0/24 subnet. Instances lying in the KVM use the IP address from the subnet of their native compute nodes. Docker containers residing in these instances use bridged IPs within the instances. One fog node has been assigned the IP address from the 10.1.10.0/24 subnet, and the other fog node has been assigned an IP address from the 172.16.221.0/24 subnet. The VMs residing in the fog nodes use the IP addresses from the subnets of their respective fog nodes. Docker containers within these VMs communicate with each other using bridge IPs. Cloud servers are connected with Quidway 7706 Huawei core switch through NSA 4600 Dell Sonic Firewall. This firewall has been implemented to stop intrusion, invasion, malicious access, and unauthorized access to the network. The fog nodes are connected with LAN and core through Huawei S5700 switches. These switches are layer-3 48 ports switches. They have the capability to operate at both layers, i.e., layer 2 (data link layer) and layer 3 (network layer). A round Robin domain name system (RR-DNS) has been set up for load balancing, load distribution, and fault tolerance for managing the user requests for service. The DNS server has been configured in the active directory domain controller. The server is deployed on a Dell Power Edge 2950 server machine with Windows Server 2016, 16 GB RAM, and 500 GB storage. The request is initiated by the user at the edge through a wireless or wired network connection. The generated request is routed to the first available fog node. This node will redirect the user’s request for service to the main managing node (VM) running with the Docker container QGIS service. In case of failure of both fog nodes, the request would be fulfilled by the core.

## 4. Results and Discussions

In the present work, different types of tests were conducted to prove that the proposed framework is resilient, i.e., continuous rendering of service, even if there is any type of failure or delay at any level.

### 4.1. Antifragility and Resiliency at the Container Level

**Figure 7:**
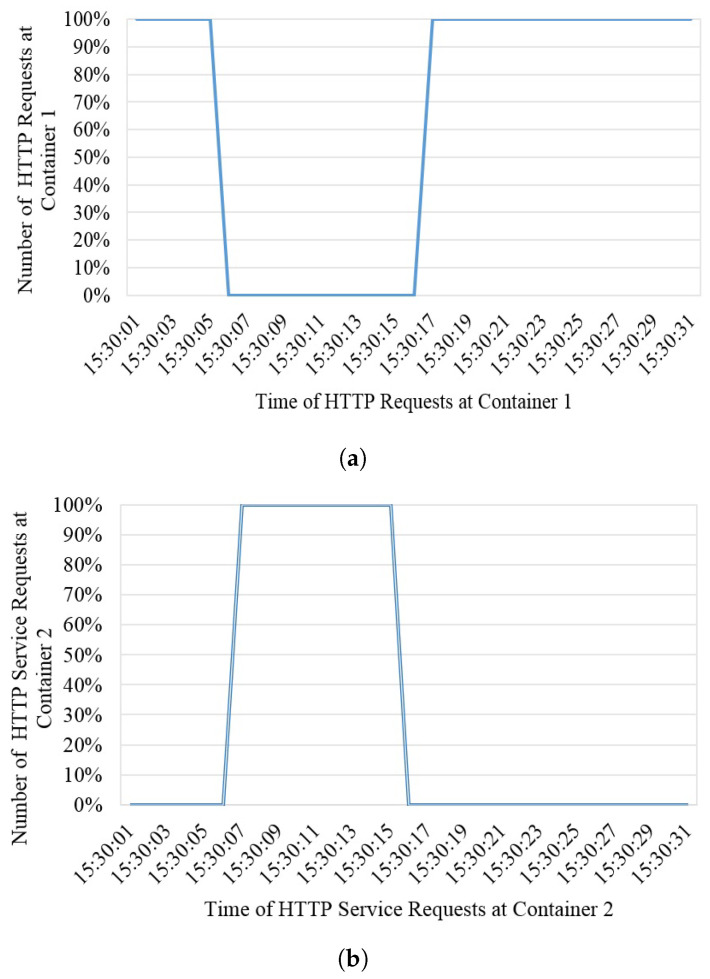
Antifragility and resiliency at the container level. (**a**) Container 1 HTTP requests, and (**b**) Container 2 HTTP request.

Figure 7 shows the anti-fragile and resilient behavior of services via containers. The QGIS was converted to a container image and hosted in a virtual machine with a dedicated network interface and port-forwarding mechanism in the host Linux machine. Therefore, two containers were deployed on one virtual machine to support each other. The proposed framework keeps an eye on the performance and availability of the containers in a virtual machine, and the above test only presents the availability of the container. The vertical graph shows the availability of the service and the horizontal values show the service status concerning time. Graphs show that as soon as the first container goes down or does not accept the incoming requests, the framework shifts all incoming requests to the second container by keeping the service status up seamlessly to the user. It happens at the same time and in the same fraction of seconds in time.

### 4.2. Antifragility and Resiliency at VM Level

**Figure 8:**
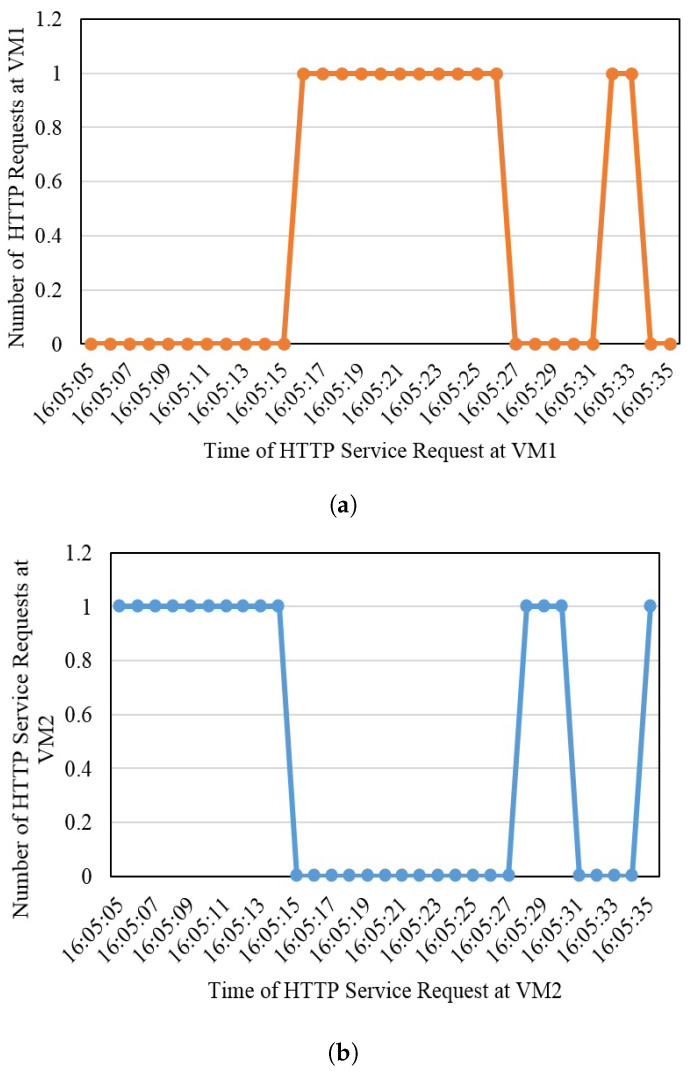
Antifragility and resiliency at VM level, (**a**) Virtual machine 1 status, and (**b**) Virtual machine 2 status.

The second test depicts the availability of virtual machines. Each virtual machine contains two containers, and when both containers are down or one virtual machine is down, incoming requests are shifted to other virtual machines in the same fog node. Time is represented on the *x*-axis, and service response with respect to time is represented on the *y*-axis. Figure 8 shows that when one virtual machine is powered off or turned down due to any known or unknown reasons, requests are shifted to other machines within no time, maintaining its antifragility and resiliency.

### 4.3. Antifragility and Resiliency at Fog Node Level

**Figure 9:**
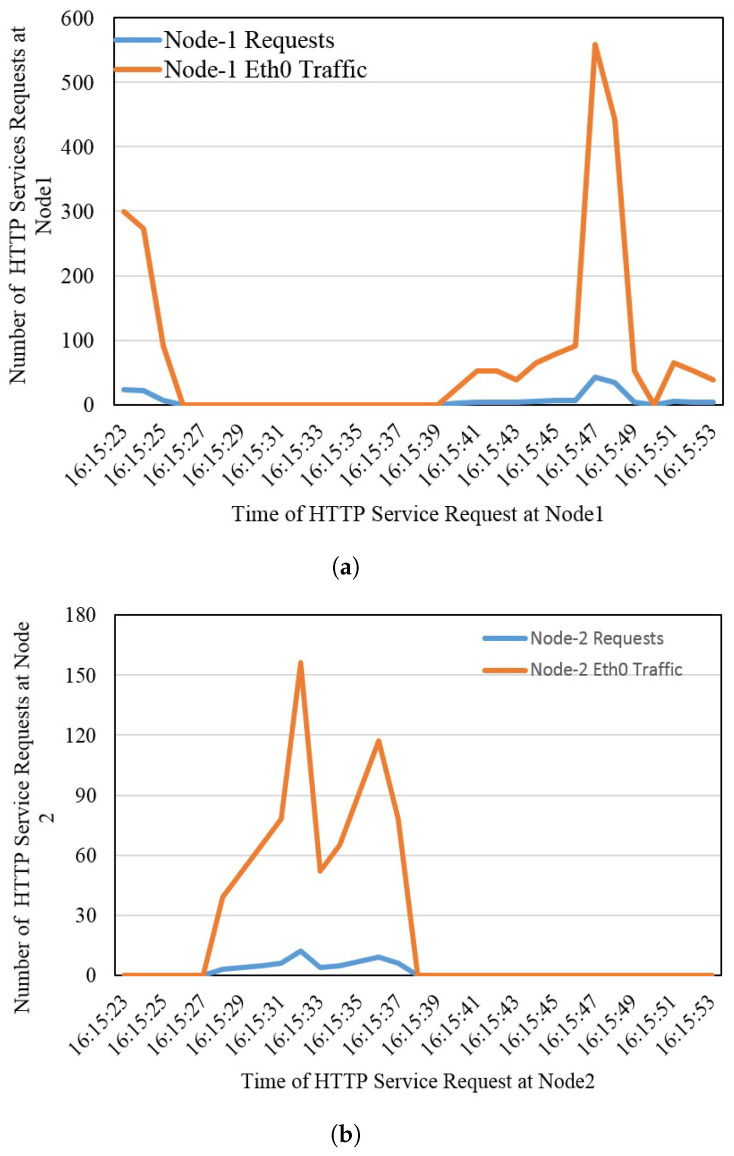
Antifragility and resiliency at the fog node level, (**a**) Fog node 1, and (**b**) Fog node 2.

Figure 9 presents the antifragility and resiliency of two peer fog nodes that are deployed in close proximity to the user. If one fog node goes down and becomes offline due to any reason, the incoming requests will be routed to its closely deployed fog node within no time to provide uninterrupted service to the users. In Figure 9, the time of receipt of service requests, the number of requests, and bandwidth consumed by the particular node are monitored, and it was observed that when fog node one is down due to any reason, incoming requests are shifted to fog node two within 1 s while keeping the user state intact. The test was repeated by turning down the fog node two, and as soon as node two was powered off, user requests were shifted back to fog node one in 1 s. There was a difference of 1 s, which is seamless to the user.

### 4.4. Requests Redirection at Serving Nodes

**Figure 10:**
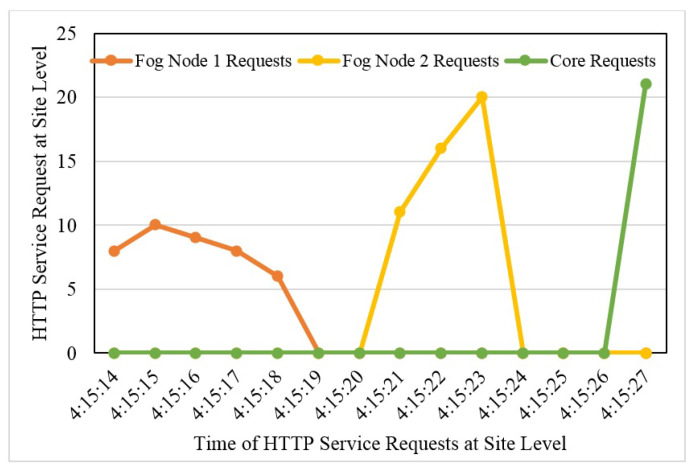
Request direction at serving nodes.

Figure 10 presents the shifting of requests from one serving node to another during downtime. The horizontal axis presents time, and the vertical axis presents the number of requests. It is clearly visible from the graphs that when Fog Node 1 is down, all requests are routed to fog node 2, and it takes 1 s to move to its peering node. One second is seamless for the user, and the service is shifted to its new node in one second. In a case when both fog node 2 and fog node 2 are down, the request has to shift to its core site that, as the graph shows, and it takes 2 s to completely shift to a new site. In the provision of services such as HTTP requests, a 2 s delay is almost unnoticeable, and the user’s session is kept intact.

### 4.5. Request Redirection Due to Overloading Nodes

**Figure 11:**
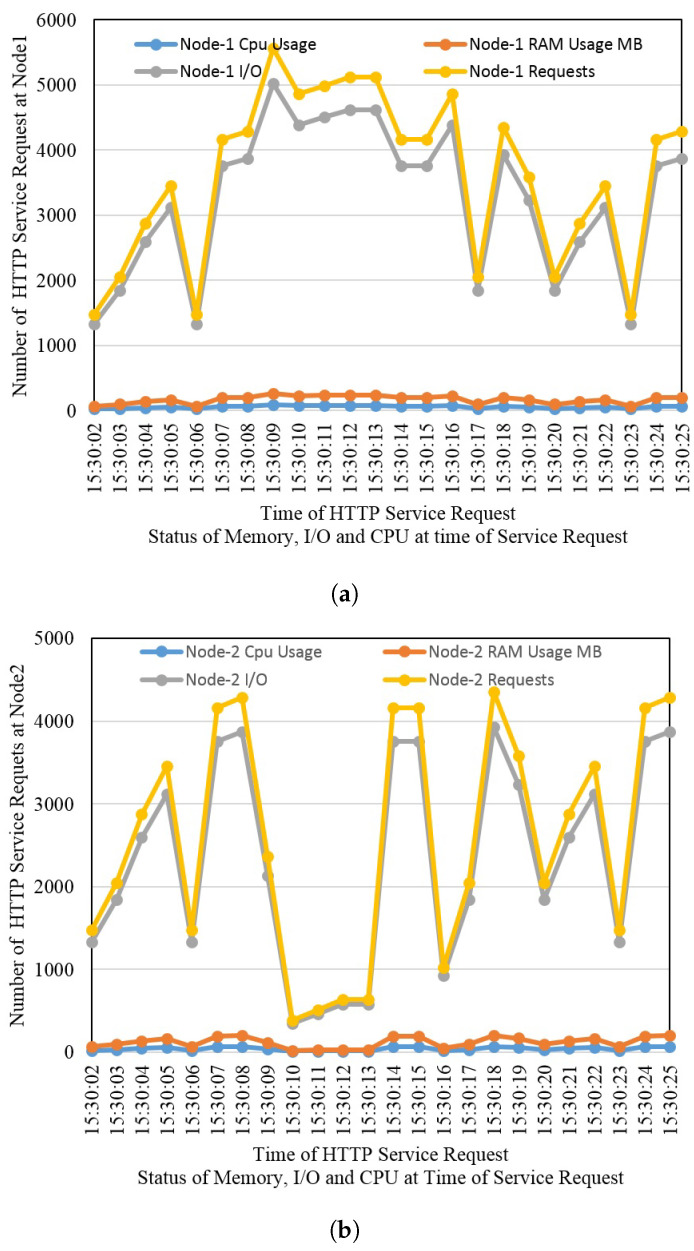
CPU, memory, and IO utilization at (**a**) Fog node 1 and (**b**) Fog node 2.

Figure 11 shows the total usage of CPU, memory, and IO operations. Containers use the resources of virtual machines, and when they are occupied by more than 50%, requests are planned to be directed to other resources. The same has been shown in the graphs as well. Upon reaching the threshold of 50% at CPU or memory, requests are shifted to the prioritized neighbor fog node. The framework keeps checking the states of all participant machines, and when resources are normalized, the incoming requests are shifted to its prioritized serving nodes.

## 5. Conclusions and Future Work

Fog computing is becoming more popular and a necessity in the coming days due to its distributed and efficient nature in accomplishing requests. It also helps to reduce the latency by reducing the large incurring cost and difficulty of deployment. Fog nodes and edge computing are integral parts of providing edge processing to users. Driverless cars, telesurgery, proactive surveillance, and proactive geofencing rely on fog technology. To provide such delicate error-free and hassle-free services to users, this work has proposed a framework at multiple fog nodes and a core platform to provide anti-fragile and resilient services to users connected at fog nodes. This framework has provided anti-fragile and resilient services by keeping the nodes up and directing the required services to its peer nodes and vice versa. The results showed a maximum of 1 s delay (unnoticeable) when a service is shifted to the core platform. No delay was recorded when the service was shifted to other containers in the same site by maintaining the user’s state as well. Finally, the guidelines for future work were proposed. The present technology is guided toward future directions for fog computing. One of the major issues is service-level agreements that have not been finalized yet. The services are inclining towards their maximum availability, and cloud services have achieved five-nines of 99.999%. Fog nodes have to remain available with consistent service availability. Cloudlets are becoming popular in fog computing as nodes. Availability, scalability, mobility, fault tolerance, QoS, bandwidth management, and artificial-intelligence-capable fog nodes are inevitable in the future. The fog has nodes that are additional middleware to access the services, algorithms, and intelligence required at fog nodes, which can decide to furnish the requests locally instead of communicating with the core platform.

## Figures and Tables

**Table 1:** Limitations of CC and FC.

Features	Fog	Cloud
Management	Distributed	Centralized
Computation device	Any device with computation power	Powerful server system
Nature of failure	Highly Diverse	Predictable
Distance from user	Close	Far
Network latency	Low	High
Node Mobility	High	Very low
No of intermediate hops	One/Few	Multiple
Application type	Latency-Aware	Non-latency aware
Real-time application handling	Achievable	Difficult
Participating nodes	Constantly dynamic	Variable
Storage capacity	Low	High

**Table 2:** Analytical summary of the literature review.

	Ref.	Approaches								Applications			
		Fault-Tolerance						Failure Induction	Anti-Fragility	Smart City	Smart Health	Smart Traffic	Vehicular Adhoc Networks
		Proactive Technique				Reactive Technique	Resiliency						
		Redundancy	Replication	Prediction	Migration								
Other infrastructure	[44]	✘	✘	✔	✘	✘	✘	✘	✘	✘	✘	✘	✘
	[19]	✘	✘	✘	✘	✔	✘	✘	✘	✘	✘	✘	✘
	[18]	✘	✘	✘	✘	✔	✘	✘	✘	✘	✘	✘	✘
Cloud computing	[24]	✔	✔	✘	✘	✘	✘	✘	✘	✘	✘	✘	✘
	[12]	✘	✘	✔	✘	✘	✘	✘	✘	✘	✘	✘	✘
	[35]	✔	✘	✘	✘	✘	✘	✘	✘	✘	✘	✘	✘
	[37]	✘	✘	✘	✔	✘	✘	✘	✘	✘	✘	✘	✘
	[1]	✘	✘	✘	✘	✘	✔	✔	✔	✘	✘	✘	✘
	[12]	✘	✘	✘	✔	✘	✘	✘	✘	✘	✘	✘	✘
	[45]	✘	✘	✘	✘	✘	✔	✔	✘	✘	✘	✘	✘
	[46]	✘	✘	✘	✘	✘	✔	✔	✔	✘	✘	✘	✘
Fog computing	[5]	✘	✘	✘	✘	✘	✘	✘	✘	✔	✔	✔	✘
	[40]	✘	✘	✘	✘	✘	✘	✘	✘	✘	✘	✘	✔
	[41]	✘	✘	✘	✘	✘	✘	✘	✘	✘	✘	✔	✘
	[47]	✘	✘	✘	✘	✘	✘	✘	✘	✘	✘	✘	✔
	[48]	✘	✘	✘	✘	✘	✘	✘	✘	✘	✔	✘	✘
	[49]	✘	✘	✘	✘	✘	✘	✘	✘	✔	✘	✘	✘

**Table 3:** Hardware used for the proposed framework.

Layer	Item	Model	Specification
Fog layer	Server Machines	2 Dell Power Edge R710	Intel Xeon 5600
	Processor	Quad Core	2.26 GHz
	Hard Disk	SATA	ITB
	RAM	DDR3	32 GB
	Server Machine	Dell Power Edge 2900	16 GB RAM, 500 GB Storage
	Network I/O	Gigabit Ethernet	2 Interfaces
	Network Switch	2 Huawei S5700	Layer 3-switch 48 port
	Network Firewall	NSA4600 Dell Sonic Wall	IPS 2.0 Gbps, Anti-Malware Inspection Throughput 1.1 Gbps 2 GB Ram, 1 GB RAM, MIPS 64 Octeon processor
	Core Switch	Quidway 7706 Huawei	
Core layer	Server Machines	Dell Power Edge R710	Intel Xeon 5600
Edge layer	Laptop, Cell Phones	Dell latitude 3450, iPhone, Android Phone	Latest

**Table 4:** Software used for the proposed framework.

Layer	Item	Description
Fog layer	Fog nodes OS	Windows Server 2016
	Cluster	Hypervisor
	Virtualization	VM, Containers
	VM OS	Ubuntu 14.04
	Containers	Docker 16.1
	Service	QGIS
	VM Converter	MVMC (Microsoft Virtual Machine Converter)
	Zabbix Server(SNMP)	Zabbix Server, Zabbix Client
	WinSCP	File Transfers using SSH
Core layer	Putty	SSh, Telnet Client
	OS	Ubuntu 14.04
	Cloud Application	Open Stack spike
	Hypervisor	KVM
	Qemu	Virtual Machine Conveter
Edge layer	OS	Windows 10, Android 9.0, iOS 12.3.1
	Browser	Edge, Internet Explorer, Safari, Mozilla Firefox
	Connection	Wireless

## Data Availability

The dataset and code is available from the corresponding author on reasonable request.
